# Slower rates of accumulation of DNA damage in leukocytes correlate with longer lifespans across several species of birds and mammals

**DOI:** 10.18632/aging.102430

**Published:** 2019-11-15

**Authors:** Kurt Whittemore, Eva Martínez-Nevado, Maria A. Blasco

**Affiliations:** 1 Telomeres and Telomerase Group, Molecular Oncology Program, Spanish National Cancer Research Centre (CNIO), Madrid E-28029, Spain; 2 Veterinary Department, Madrid-Zoo Aquarium, Madrid 28011, Spain

**Keywords:** species, lifespan, DNA damage, short telomeres

## Abstract

Although there is previous evidence showing an increase in various types of DNA damage with aging in mice and humans, a comparative study determining accumulation rates of DNA double strand breaks, as determined by presence of phosphorylated histone H2AX (γH2AX), in leukocytes of individuals of different ages from phylogenetically distinct species from birds to mammals was lacking. Here, we demonstrate that the rate of accumulation of DNA damage as measured by the DNA damage marker γH2AX correlates with species longevity in dolphins, goats, reindeer, American flamingos, and griffon vultures. In particular, we find that species that show slower rates of accumulation of the DNA damage marker γH2AX also live longer.

## INTRODUCTION

Different species have very different lifespans ranging from less than 1 day for mayflies to more than 400 years for the Greenland shark [1, 2]. However, the exact cause of these differences in longevity are still largely unknown. Our group recently showed that the rate of telomere shortening with age correlates with lifespan in a variety of species from birds to mammals [3]. Species with very fast telomere shortening rates such as mice have very short lifespans, and species with very slow telomere shortening rates such as humans have very long lifespans [3, 4]. It is interesting to note that species that share a similar longevity in spite of being evolutionarily distant like flamingos and elephants, also show a similar rate of telomere shortening, while evolutionarily closer species like mice and elephants, show very different longevities and also have very different rates of telomere shortening [3]. These findings suggest that longevity can be determined, at least in part, by epigenetic traits, such as the rate of telomere shortening. Furthermore, these findings pose the interesting question of which is the molecular determinant by which higher telomere shortening rates lead to shorter longevities. An obvious answer is that higher rates of telomere shortening will be associated to faster accumulation of critically short/dysfunctional telomeres, which are known to contribute to activation of a persistent DNA damage response stemming from telomeres, which leads to loss of cell viability and aging phenotypes [5, 6]. Thus, species that shorten telomeres at faster rates will reach telomere exhaustion and trigger a persistent DNA damage response earlier than those species that are able to maintain telomeres protected for a longer period of time. A short/dysfunctional telomere is recognized by the cell as an irreparable DNA double strand break (DSB), triggering a persistent DNA damage response which results in phosphorylation of γH2AX, and which eventually leads to cell death and/or senescence [7, 8]. In turn, induction of cellular senescence either owing to critically short telomeres or to other insults is also associated with increased γH2AX levels, involving in some instances the mTOR pathway [9–11]. Thus, accumulation of cells with DNA damage throughout lifespan should also correlate with species longevity.

DNA damage has been proposed to be one of the major determinants of aging and lifespan [12–19]. In particular, the abundance of the 8-oxo-deoxyguanosine lesion in mitochondrial and nuclear DNA was previously found to correlate inversely with species lifespan [18, 19], indicating that lower levels of this type of DNA damage are associated with longer lifespans. Strikingly, the correlation between 8-oxo-deoxyguanosine levels and species lifespan followed a power law curve [18], similar to the power law relationship that was recently reported by us between telomere shortening rates and species lifespan [3].

**Figure 1:**
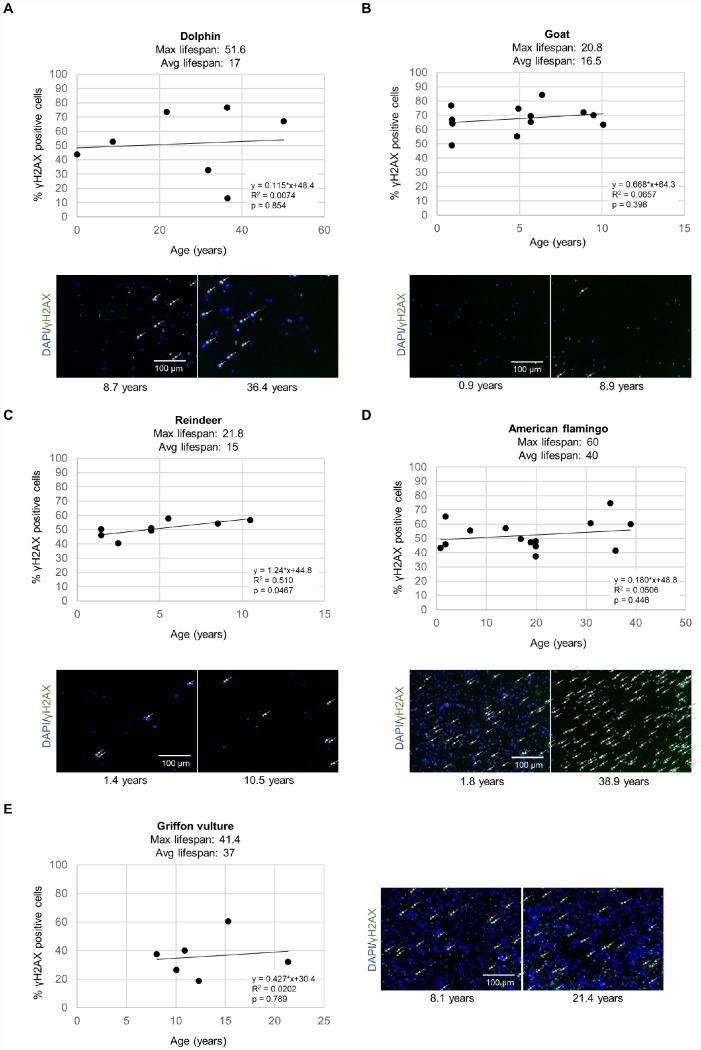
**DNA damage γH2AX measurements for various species.** The level of γH2AX was measured by immunofluorescence in leukocytes in a high-throughput manner in individuals of different ages for (**A**) bottlenose dolphins (*Tursiops truncatus*), (**B**) goats (*Capra hircus*), (**C**) reindeer (*Rangifer tarandus*), (**D**) American flamingos (*Phoenicopterus ruber*), (**E**) griffon vultures (*Gyps fulvus*). Each point represents the values for a different individual. The correlation coefficient (R^2^), slope (rate of γH2AX increase in % positive cells per year), and y-intercept are presented on the graphs. Representative images show cell nuclei stained with DAPI in blue and yH2AX stain in green for a young individual and an older individual for each species. White arrows indicate γH2AX positive cells.

However, the rates of accumulation of global DNA damage with aging have not been previously measured in parallel in a wide variety of phylogenetically distinct mammalian and bird species using the same techniques to allow for direct comparisons. Here, we set to evaluate whether the rates of accumulation of global nuclear DNA damage as determined by the DNA damage marker γH2AX correlates with species lifespan of phylogenetically distant species. To this end, we measured the percentage of cells with DNA damage as determined by the intensity of the γH2AX DNA damage marker in leukocytes from individuals of different ages from several species of birds and mammals, as well as turtles, in parallel. The DNA damage marker γH2AX was chosen since histone H2AX becomes phosphorylated to γH2AX as an early event when DNA double-strand breaks occur, and γH2AX is often used in assays for detection of DNA damage [20–23]. In particular, we measured the percentage of cells positive for the DNA damage marker γH2AX in bottlenose dolphins (*Tursiops truncatus*) of ages ranging between 1 day and 50.1 years (Figure 1A), goats (*Capra hircus)* of ages ranging between 310 days and 10.1 years (Figure 1B), reindeer (*Rangifer tarandus*) of ages ranging between 1.44 and 10.5 years (Figure 1C), American flamingos (*Phoenicopterus ruber*) of ages ranging between 288 days and 38.9 years (Figure 1D), and griffon vultures (*Gyps fulvus*) of ages ranging between 8.06 and 21.4 years (Figure 1E). We also included three loggerhead sea turtles (*Caretta caretta*) of ages ranging between 8.35 and 43.7 years, a very long-lived reptile, as we noticed that they had extremely intense telomere signals as determined by quantitative telomere FISH or Q-FISH (Supplementary Figure 1A, 1B) [24]. The telomere lengths and the percent short telomeres of the loggerhead sea turtles at different ages are shown in Supplementary Figure 1B. For comparison, we also show here telomere length at different ages in the griffon vulture and the American flamingo (data obtained from a previous publication [3]). In particular, the telomere length for the sea turtle ranged from approximately 80-120 kb, whereas the vultures and flamingos had telomere lengths in the 15–25 kb range (Supplementary Figure 1B). Owing to the fact that we only had three turtles, we included the data in separate graphs from those of the rest of the species in the different comparisons (Supplementary Figure 1B).

For DNA damage quantification, we performed immunofluorescence analysis with an antibody against γH2AX to detect DNA damage, including detection of double strand DNA breaks and of critically short telomeres, and quantified fluorescence in a high throughput manner in 384 well plates as described previously [21, 25]. A cell was considered to be positive for the DNA damage marker γH2AX if the pan-nuclear fluorescence intensity value per nuclei was higher than a threshold set at the 50^th^ percentile of the intensity values from a young sample for that species as described previously [21, 25]. In general, all species showed an increase in % of cells with DNA damage with increasing age (Figure 1). Note that each data point in the figure represents the data from a different individual in a cross-sectional study (Figure 1).

Next, by using these data, we calculated the rate of increase of % of cells with DNA damage with aging. Bottlenose dolphins showed a rate of γH2AX increase of 0.115% positive cells/year (Figure 1A). Goats showed a rate of γH2AX increase of 0.668% positive cells/year (Figure 1B). Reindeer showed a rate of γH2AX increase of 1.24% positive cells/year (Figure 1C). American flamingos showed a rate of γH2AX increase of 0.180% positive cells/year (Figure 1D). Griffon vultures showed a rate of γH2AX increase of 0.427% positive cells/year (Figure 1E). Loggerhead sea turtles showed a very slow rate of γH2AX increase, of 0.137% positive cells/year (Supplementary Figure 1A).

**Figure 2:**
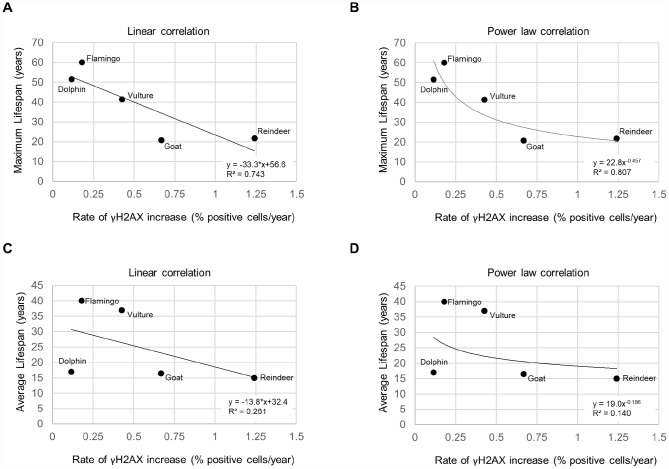
**Lifespan vs. rate of γH2AX increase.** (**A**) Maximum lifespan vs rate of γH2AX increase fit with a linear regression line. (**B**) Maximum lifespan vs rate of γH2AX increase fit with a power law regression line. (**C**) Average lifespan vs rate of γH2AX increase fit with a linear regression line. (**D**) Average lifespan vs rate of γH2AX increase fit with a power law regression line.

We next investigated the relationship between the rate of increase of % of cells positive for the γH2AX DNA damage marker and species lifespan. For the species maximum lifespan, we used the AnAge database [2]. The average lifespans were obtained from various sources (Supplementary Table 1). We observed a trend for shorter species lifespans with higher rates of accumulation of cells with DNA damage (Figure 2). A graph of the maximum lifespan vs the rate of γH2AX increase resulted in an R^2^ value of 0.743 with a linear trendline (Figure 2A), and an R^2^ value of 0.807 with a power law trendline (Figure 2B). A graph of the average lifespan vs the rate of γH2AX increase resulted in lower R^2^ values with an R^2^ value of 0.261 with a linear trendline (Figure 2C), and an R^2^ value of 0.140 with a power law trendline (Figure 2D). These findings show a very good correlation between maximum lifespan and the rate of increase of cells with DNA damage per year both when using a linear and a power law fit to the data.

**Figure 3:**
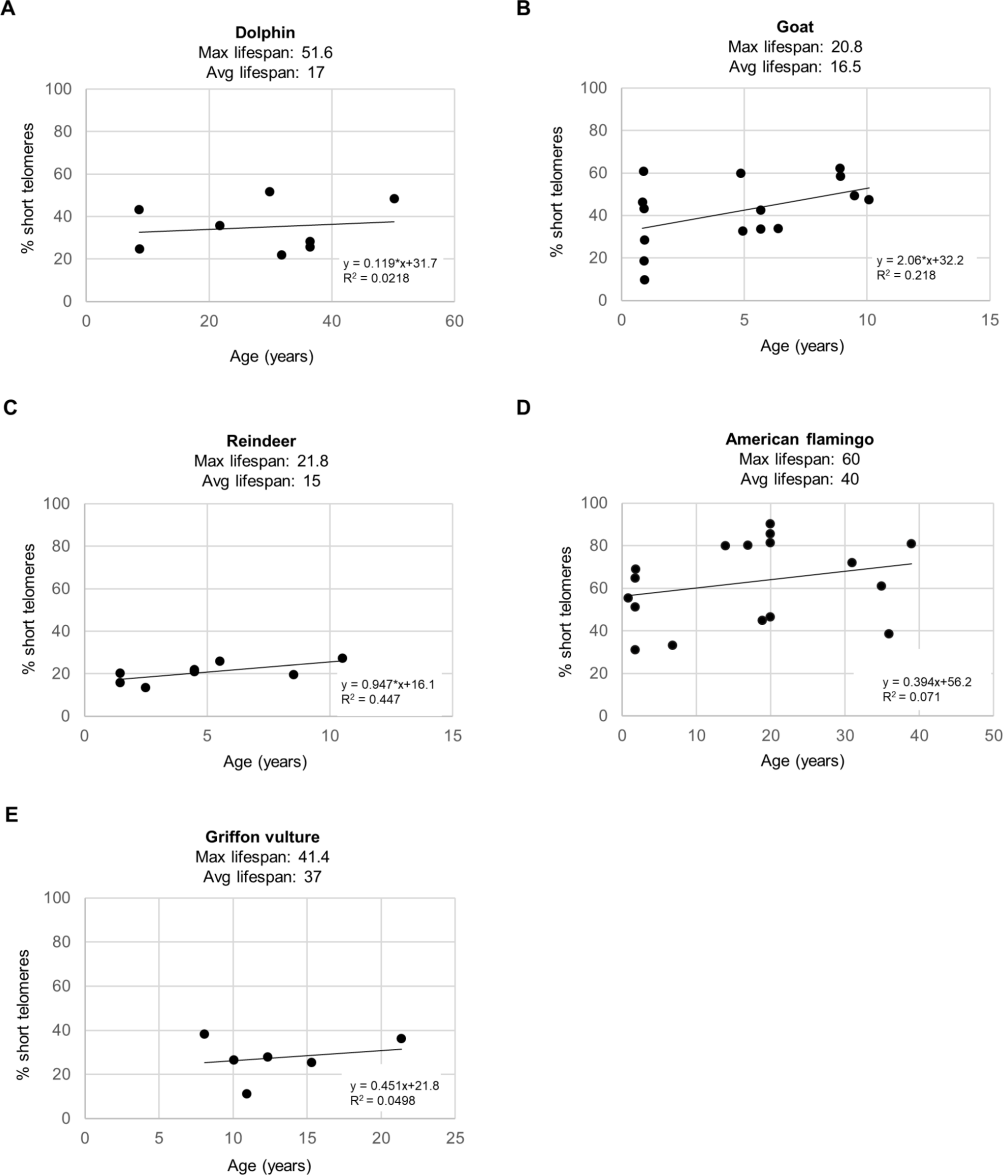
**Percent short telomeres of various species.** The percentage of short telomeres was measured by HT Q-FISH in a high-throughput manner in individuals of different ages for (**A**) bottlenose dolphins (*Tursiops truncatus*), (**B**) goats (*Capra hircus*), (**C**) reindeer (*Rangifer tarandus*), (**D**) American flamingos (*Phoenicopterus ruber*), and (**E**) griffon vultures (*Gyps fulvus*). Each point represents the values for a different individual. The correlation coefficient (R^2^), slope (rate of increase of % short telomeres per year), and y-intercept are presented on the graphs. The percentage of short telomeres was calculated by a threshold which corresponds to the 25^th^ percentile of the telomere fluorescence intensity values of a young sample for a given species.

**Figure 4:**
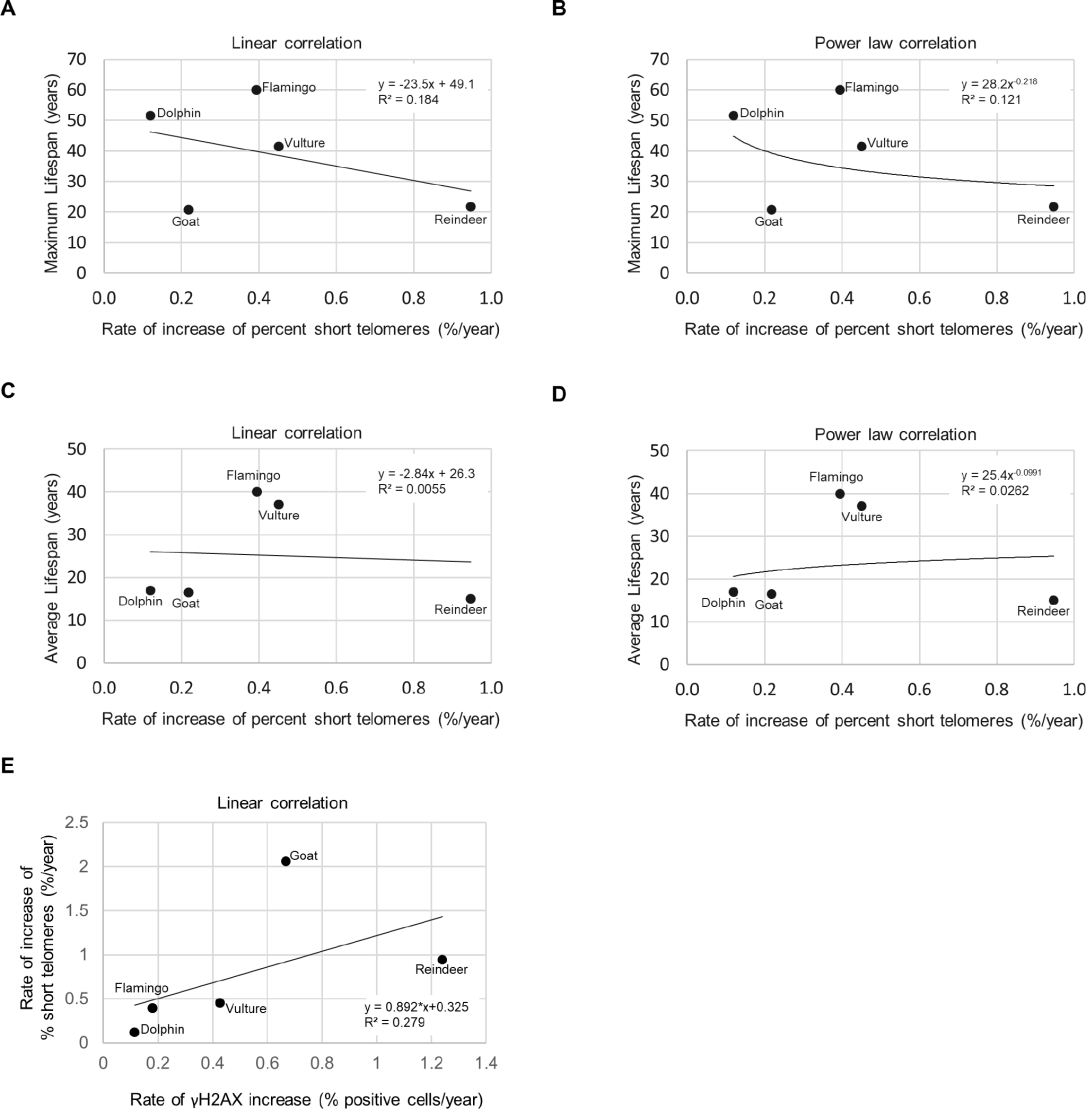
**Lifespan, short telomeres, and DNA damage.** (**A**) Maximum lifespan vs rate of increase of % short telomeres fit with a linear regression line. (**B**) Maximum lifespan vs rate of increase of % short telomeres fit with a power law regression line. (**C**) Average lifespan vs rate of increase of % short telomeres fit with a linear regression line. (**D**) Average lifespan vs rate of increase of % short telomeres fit with a power law regression line. (**E**) Rate of increase of percentage of short telomeres vs. rate of γH2AX increase.

As short dysfunctional telomeres may be contributing to increased DNA damage with aging, we also determined the percentage of cells with very short telomeres by using high-throughput quantitative telomere FISH or HT Q-FISH in dolphins (Figure 3A), goats (Figure 3B), reindeers (Figure 3C), American flamingos (Figure 3D), and griffon vultures (Figure 3E) [24]. The percentage of short telomeres was calculated by a threshold which corresponds to the 25^th^ percentile of the telomere fluorescence intensity values of a young sample for a given species [24, 26]. As expected, all species showed an increase in the percentage of short telomeres with age, concomitant with the fact that telomere length decreased with age as shown in a previous publication [3]. A graph of the maximum lifespan vs the rate of increase of percent short telomeres resulted in an R^2^ value of 0.184 with a linear trendline (Figure 4A), and an R^2^ value of 0.121 with a power law trendline (Figure 4B). A graph of the average lifespan vs the rate of increase of percent short telomeres resulted in lower R^2^ values with an R^2^ value of 0.0055 with a linear trendline (Figure 4C), and an R^2^ value of 0.0262 with a power law trendline (Figure 4D). Thus, a modest correlation was obtained between maximum lifespan and the rate of increase of short telomeres. Again, as observed with DNA damage, comparisons with average lifespan were weaker. These findings suggest that short telomeres could be contributing to the increase in DNA damage that we detect associated with aging. In agreement with this, we also found a modest correlation between the rate of increase of short telomeres and the rate of increase of DNA damage with an R^2^ value of 0.279 (Figure 4E), in agreement with the presence of short telomeres being partially causative of increased DNA damage with age. In support of this, we noticed that the species with the highest rates of γH2AX increase (>0.6 % positive cells/year) were the species with the highest rates of increase in percentage of short telomeres (>0.5 %/year) (Figure 4E).

We also determined how the rate of γH2AX increase and the rate of increase of percent short telomeres variables performed in a multivariate linear regression model to predict the average or maximum species lifespan (Supplementary Tables 2–4). A limitation of this analysis is that we could only include 5 species for which we had both telomere shortening rates and DNA damage rates. The data input into the model is displayed in Supplementary Table 2. The model showed that the variables could not predict the species average lifespan with a p-value of 0.848 (Supplementary Table 3) or maximum lifespan with a p-value of 0.125 (Supplementary Table 4). Note that the maximum lifespan model p-value was more significant than the average lifespan model p-value, just as a correlation between rate of γH2AX increase and maximum lifespan had a higher R^2^ value (R^2^ = 0.807; Figure 2B) than the correlation between rate of γH2AX increase and average lifespan (R^2^ = 0.140; Figure 2D) with these 5 species. The model also indicated that the rate of γH2AX increase was a more significant variable (p=0.358) than the rate of increase of percent short telomeres for predicting lifespan (p=0.447) (Supplementary Table 4). More significant results may be expected if additional species and more individuals per species could be included in the analysis. Nevertheless, the observed trends are of interest since the R^2^ between the rate of yH2AX increase and maximum lifespan was above 0.7, similar to that shown in other studies [18].

**Figure 5:**
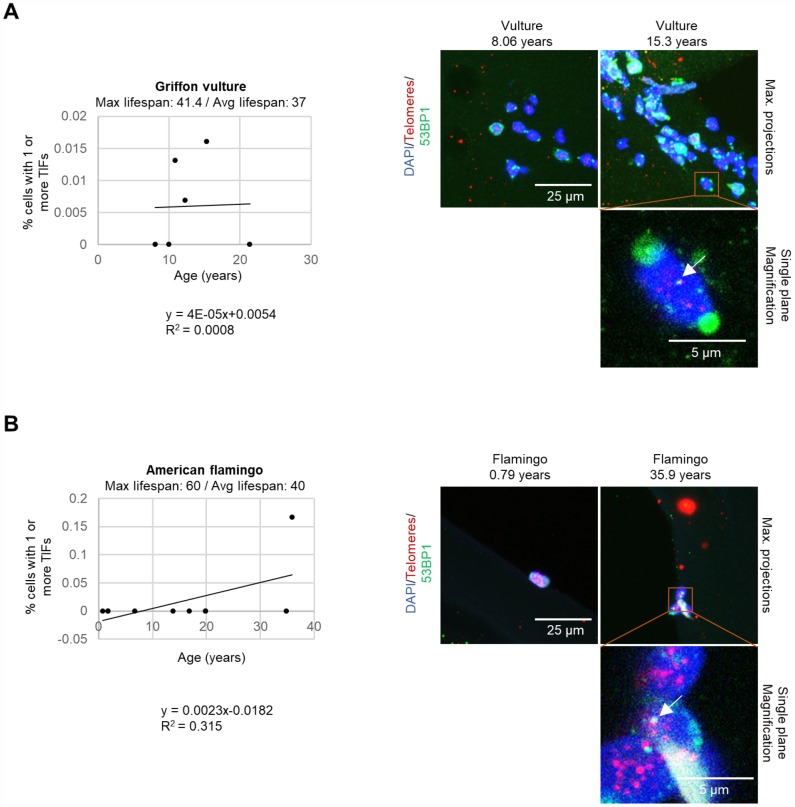
**TIFs measured in leukocytes.** (**A**) The percentage of cells with one or more TIFs in griffon vultures. The age and number of cells measured for each individual was as follows: 8.06 years: 77 cells, 10 years: 29 cells, 10.9 years: 153 cells, 12.3 years: 146 cells, 15.3 years: 125 cells, and 21.4 years: 173 cells. (**B**) The percentage of cells with one or more TIFs in American flamingos. The age and number of cells measured for each individual was as follows: 0.79 years: 3 cells, 1.75 years: 12 cells, 1.8 years: 34 cells, 6.75 years: 21 cells, 13.8 years: 11 cells, 16.9 years: 9 cells, 19.9 years: 7 cells, 19.9 years: 7 cells, 34.9 years: 20 cells, and 35.9 years: 6 cells. In the representative images, the nuclei are stained blue with DAPI, the telomeres are red, and the 53BP1 stain is in green. 53BP1 staining at the very edge of nuclei was not counted as foci. The top row of the representative images shows maximum projections which are the result of taking the maximum value of several different z-planes. The magnification image is displayed for a single plane rather than a maximum projection to show co-localization of the stains. A white arrow indicates a colocalization of 53BP1 and a telomere spot (a TIF).

We next set to study whether the increase of DNA damage could be located specifically at telomeres, the so-called telomere induced DNA damage foci or TIFs. To this end, we used a small group of species and determined the co-localization of the DNA damage marker 53BP1 with telomeric DNA by performing an immuno-telomere-FISH experiment [26]. A graph of the percent of cells with one or more TIFs (telomere induced foci) is shown for griffon vultures (Figure 5A), American flamingos (Figure 5B), and loggerhead sea turtles (*Caretta caretta*) (Supplementary Figure 1C). Although very few cells with TIFs were detected, we observed that individuals with increased TIFs (telomere induced foci) were the older individuals. We were only able to obtain samples from 3 individual loggerhead sea turtles, but we observed that only the oldest turtle at an age of 43.7 years old had TIFs (Supplementary Figure 1C), although we did not see increased global DNA damage in the turtle.

**Figure 6:**
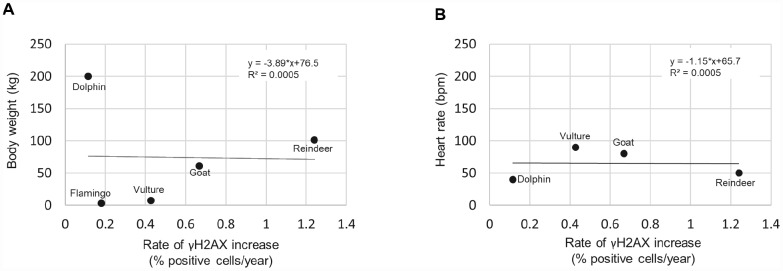
**Rate of γH2AX increase correlated with species body weight and heart rate.** (**A**) Body weight vs rate of γH2AX increase. (**B**) Heart rate vs rate of γH2AX increase.

Finally, previous studies have correlated other variables with species lifespan such as body weight [27], and heart rate [28, 29]. However, we did not find any of these variables to correlate with the rate of γH2AX increase (Figure 6), at least with the number of species that were included. A graph of the body weight vs rate of γH2AX increase resulted in an R^2^ of 0.0005 (Figure 6A). A graph of the heart rate vs the rate of γH2AX increase resulted in an R^2^ of 0.0005 (Figure 6B). Therefore, with this dataset, we did not detect a strong correlation between the rate of γH2AX increase and body weight or heart rate variables.

## CONCLUSIONS

Here, we find that increased global rates of DNA damage, as determined by the DNA damage marker γH2AX which detects occurrence of double stranded DNA breaks in the genome, inversely correlates with species longevity, and these correlations fit both a linear and a power law curve. In particular, we determined here the rates of increase of the DNA damage marker γH2AX in leukocytes of phylogenetically distant species of birds and mammals in parallel and using the same experimental method. Previous studies have also shown a correlation between certain types of DNA damage and aging [12, 13, 15, 16, 18, 19]. Among primate species, those with a better ability to repair UV-induced double stranded DNA damage were also shown to have longer lifespans [30, 31]. In the case of rodents, rodent species with higher DNA repair rates were also shown to have a longer lifespans [32]. Of particular interest to the findings described here, an inverse correlation between levels of mitochondrial and nuclear 8-oxo-deoxyguanosine in several different species [18, 19] was described to follow a power law relationship, similar to the power law relationship recently reported by us for telomere shortening rates and species lifespan [3] and to what we describe here for rates of the γH2AX DNA damage marker and species lifespan.

Indeed, DNA damage accumulation with aging and telomere shortening may be related processes. Critically short telomeres as the result of cell proliferation throughout life to repair damaged tissues trigger a DNA damage signal specifically at telomeres, the so-called telomere induced DNA damage foci, or TIF, which are characterized by presence of the DNA damage marker yH2AX at telomeres [8, 33, 34]. In turn, some types of DNA damage such as UV irradiation or oxidative stress can lead to telomere shortening [35–37]. Similarly, higher levels of nuclear 8-oxo-deoxyguanosine have been shown to correlate with shorter telomeres [36]. Future studies warrant studying how different types of DNA damage correlate, and how this DNA damage correlates with telomere shortening rates.

Our results also indicate that there is a slightly better correlation when using a power law model rather than a linear model when plotting species maximum lifespan and rate of DNA damage. As more individual animals and species are added to these plots in future studies, the nature of the mathematical trend will likely become more apparent.

We also measured the percentage of short telomeres of the species in this study, and we found that all of the species showed an increase in the percentage of short telomeres with age. This result is concomitant with the fact that average telomere length shortens with age in many species [3, 4, 38–42]. Several studies have suggested that the percentage of short telomeres is more indicative of health and senescence than average telomere length [4, 5]. The percentage of short telomeres is an important metric since it is the length of the shortest telomere in a cell that induces a DNA damage response and cell senescence rather than the average telomere length of the telomeres on all of the chromosomes [5, 43]. Here we also noticed a mild trend for species with longer maximum lifespans to have a lower rate of increase of percent short telomeres, thus accumulating short telomeres more slowly with age. We also observed that species with the highest rates of γH2AX increase have the highest rates of increase of percent short telomeres with age. These results make a connection between γH2AX DNA damage, short telomeres, and lifespan. As cells accumulate DNA damage and short telomeres, they will enter into a state of senescence, thus accelerating the aging process and shortening lifespan.

## MATERIALS AND METHODS

### Blood samples

Blood samples were obtained from the Madrid zoo. Only one timepoint was measured for each individual so this is a cross-sectional study. For dolphins (*Tursiops truncatus*), blood was sampled from 9 individuals with an age range from 8.6 years to 50.1 years. For goats (*Capra hircus*), blood was sampled from 15 individuals with an age range from 0.85 years to 10.1 years. For reindeer (*Rangifer tarandus*), blood was sampled from 8 individuals with an age range from 1.44 years to 10.5 years. For American flamingos (*Phoenicopterus ruber*), blood was sampled from 15 individuals with an age range from 0.79 years to 50.1 years. For the griffon vulture species (*Gyps fulvus*), blood was sampled from 6 individuals with an age range from 8.06 years to 21.4 years. For the Sumatran elephant species (*Elephas maximus sumatranus*), blood was sampled from 4 individuals with an age range from 6.14 years to 24.7 years. The blood samples were processed with erythrocyte lysis buffer (Qiagen cat no 79217) according to the manufacturer’s protocol. Therefore, for all species the measurements were acquired in leukocytes. The samples were then frozen at -80 °C slowly in a Nalgene Cryo Freezing Container (Nalgene Cat no 5100-0001).

### HT immunofluorescence for γH2AX DNA damage

High-throughput immunofluorescence for γH2AX was performed in a 384 well plate and images were captured with an Opera High Content Screening System as described in the HT microscopy section. First, frozen erythrocyte lysis buffer processed blood samples were thawed quickly and resuspended in complete RPMI media. Cells were attached to the wells (30,000–150,00 cells/well of clear-bottom black-walled 384-well plates (CellCarrier-384 Black Optically Clear Bottom plates Cat No 6007550) which had been precoated with 0.001% weight/volume (poly)L-lysine solution (Sigma P8920-100 mL) for 30 min at 37 °C. The wells on the outer edge of the plate were not used. The cells were incubated for 37 °C for no more than 4 hours before fixation. The cells were then fixed for 10 min by adding formaldehyde to the media so that the final concentration of formaldehyde was 4%. The cells were washed 3X with TBS (Tris-Buffered Saline) 5 min, permeabilized with 0.5% Triton X-100 in TBS 10 min, washed 3X with TBS 5 min, and then blocked by adding FBS. The plate was incubated for 2 hr at room temperature. After the blocking, the primary antibody (1:1000 Cell Signaling Phospho-Histone H2A.X (Ser139) (20E3) Rabbit mAb Cat No 97185) in 5% BSA in PBS was added, and the plate was incubated overnight at 4 °C. The next day, the primary antibody solution was removed, the wells were washed 3X with TBS 5 min, the secondary antibody (Invitrogen Goat anti-Rabbit IgG (H+L) Cross-Adsorbed Secondary Antibody, Alexa Fluor 488 Cat No A11008) diluted in 5% BSA in PBS was added, the plate was incubated 1 hr at room temperature, the wells were washed 3X with TBS 5 min, the wells were washed 1X with TBS containing 1 μg/mL DAPI (4′,6-diamidino-2-phenylindole) to stain the nuclei, the plate was washed 1X with TBS 5 min, the TBS was removed and 50 μL of Mowiol solution (10 g mowiol (polyvinyl alcohol; Calbiochem Cat no 475904), 25 mL glycerol, 25 mL H_2_0, 12 mL 0.2 M Tris HCl pH 8.5, and 2.5% w/v DABCO (1,4-Diaza [2.2.2] bicyclooctane; Sigma-Aldrich Cat no D27802-25G)) was added. The plates were then covered with aluminum foil lids to block light and stored at 4 °C in the dark until imaging as described in the HT microscopy section.

### HT microscopy

Images for high-throughput (HT) experiments were acquired on an Opera High Content Screening System (PerkinElmer, Inc.) equipped with a UV lamp, 488 nm laser, and a 40X/0.9NA water-immersion objective. Images were analyzed with Acapella Image analysis software (PerkinElmer, Inc.). Data was analyzed with Microsoft Excel (Microsoft). A cell was considered to be positive for the DNA damage marker γH2AX if the pan-nuclear fluorescence intensity value per nuclei was higher than a threshold set at the 50^th^ percentile of the intensity values from a young sample for that species.

### Immuno-telomere-FISH

Leukocytes were first fixed in a paraffin block. This was accomplished by centrifuging at 1500 rpm 5 min, removing the supernatant, and resuspending in 50 μL 10% formaldehyde buffered at pH 7.0 for 20 min. Next 10% gelatin from porcine skin (Sigma Cat no G1890-100G) in PBS (phosphate buffered saline solution) was microwaved for a short time, and 50 μL of this gelatin solution was added to the sample. The sample was then incubated at 4 °C 5 min and 1 mL of 10% formaldehyde buffered at pH 7.0 was added. The sample was then incubated overnight at 4 °C. The next day, the liquid was removed, the bottom of the tube was cut, and the solidified gel was pushed into a cassette. The CNIO histopathology core then made a paraffin block and cut paraffin sections onto glass slides for staining.

The telomeres were stained and the 53BP1 was stained with the following protocol, which has also been described previously [26]. Glass slides with paraffin sections were deparaffinized by the CNIO histopathology core. The slides were washed 2X5 min in PBS, permeabilized with 0.5% Triton X-100 in PBS for 3 hr, washed 3X5 min in PBS, fixed in 4% formaldehyde in PBS for 2 min, washed 3X5 min in PBS, washed with 70% ethanol for 5 min, washed with 90% ethanol for 5 min, washed in 100% ethanol 5 min, and then air dried overnight. After air drying the slides, 30 μL of telomere probe mix (10 mM TrisCl pH 7, 25 mM MgCl_2_, 9 mM citric acid, 82 mM Na_2_HPO_4_, 70% deionized formamide (Sigma), 0.25% blocking reagent (Roche), and 0.5 μg/mL Telomeric PNA probe (Panagene)) was added to each slide. A cover slip was applied, and the slides were incubated for 3 min at 85 °C. The slides were then incubated 2 hr at room temperature in a wet chamber in the dark. Slides were washed 2X15 min in 10 mM TrisCl pH 7, 0.1% BSA in 70% formamide with shaking, and washed 2X5 min in PBS. Next the immunofluorescence part to stain the 53BP1 was started by blocking the slides with 100% FBS (fetal bovine serum) for 1 hr in a humidity chamber. Next extra liquid was removed and then 50 μL of 1:500 primary antibody Novus rabbit pAb anti-53BP1 (Cat no NB100-304) was added. The slides were then incubated overnight at 4 °C. The slides were then washed 3X10 min with 0.1% Tween 20 in PBS at RT (room temperature), washed 5 min with PBS, and then 50 μL of the secondary antibody Invitrogen goat anti-rabbit IgG (H+L) AF488 (Cat no A11008) was added. The slides were covered with a glass coverslip and incubated for 1 hr at RT in a humidity chamber. The slides were then washed 3X10 min with PBS, washed 5 min with DAPI stain, washed 5 min with PBS, and then 30 μL of Vectashield was added to the slides. The slides were covered with a glass coverslip, sealed with nail polish, and stored at 4 °C until capturing images with a microscope. Confocal images were acquired as stacks using an SP5-WLL confocal microscope (Leica) and maximum projection images were created using the Fiji version of the ImageJ software (NIH) [44, 45]. We then searched for TIFs manually using the LAS AF Lite software (Leica Microsystems) by looking through each plane of the images to find co-localization of a telomere spot and a 53BP1 spot.

### HT Q-FISH

The procedure for HT Q-FISH to measure telomeres is described fully in another publication [3].

### Abundance of very old individuals in different species

We defined very old as the age above the value of 70% of the maximum lifespan for each species. For humans this would correspond to an age of 122.5*0.7 = 73.5 years old. In our study, the number of old individuals (age greater than 70% of the maximum lifespan) sampled for each species is as follows: 3/8 (37.5%) for dolphin, 0/15 (0%) for goat, 0/8 (0%) for reindeer, 0/16 (0%) for American flamingo, 0/6 (0%) for griffon vulture, and 0/3 (0%) for loggerhead sea turtle.

### Data analysis

Graphs were created and data analysis was performed in Microsoft Excel. Multivariate linear regression was performed in the R statistics software [46].

## Supplementary Material

Supplementary References

Supplementary Figure 1

Supplementary Tables
