# Supplementary material for: Slower rates of accumulation of DNA damage in leukocytes correlate with longer lifespans across several species of birds and mammals
Source: Aging (Albany NY). 2019 Nov 15;11(21):9829–45. doi: 10.18632/aging.102430 (PMC6874430; doi:10.18632/aging.102430)
Supplement: Supplementary References [file aging-11-102430-s001.pdf]

SUPPLEMENTARY REFERENCES

1. Whittemore K, Vera E, Martínez-Nevado E, Sanpera C, Blasco MA. Telomere shortening rate predicts species life span. Proc Natl Acad Sci U S A. 2019; 116:15122–15127. https://doi.org/10.1073/pnas.1902452116 PMID:31285335
2. de Magalhães JP, Budovsky A, Lehmann G, Costa J, Li Y, Fraifeld V, Church GM. The Human Ageing Genomic Resources: online databases and tools for biogerontologists. Aging Cell. 2009; 8:65–72. https://doi.org/10.1111/j.1474-9726.2008.00442.x PMID:18986374
3. SPECTOR WS. Handbook of biological data. Handb Biol data. 1956.
4. Cuyler C, Østergaard JB (2005) Fertility in two West Greenland caribou Rangifer tarandus groenlandicus populations during 1996/97: potential for rapid growth. Wildlife Biol. 2005; 11:221–228. https://doi.org/10.2981/0909-6396(2005)11[221:FITWGC]2.0.CO;2
5. James CR, Judge DS. Longevity records: life spans of mammals, birds, amphibians, reptiles, and fish (Odense University Press). 2000.
6. Wilson DE, Mittermeier RA, Cavallini P. Handbook of the mammals of the world Vol. 2: Hoofed Mammals (Lynx Edicions). 2011.
7. Hoyo J del., Elliott A, Sargatal J, Cabot J. Handbook of the birds of the world Vol. 2: New World Vultures to Guineafowl (Lynx Edicions). 1st Ed. 1994.
8. Hersh SL, Odell DK, Asper ED. Bottlenose dolphin mortality patterns in the Indian/Banana River system of Florida. 1990. pp 155–164. https://doi.org/10.1016/B978-0-12-440280-5.50012-3
9. Odell DK. Status and aspects of the life history of the bottlenose dolphin, Tursiops truncatus , in Florida. J Fish Res Board Canada. 1975; 32:1055–1058. https://doi.org/10.1139/f75-124
10. Stolen MK, Barlow J. A model life table for bottlenose dolphins (Tursiops truncatus) from the Indian river lagoon system, Florida, U.S.A. Mar Mammal Sci. 2003; 19:630–649. https://doi.org/10.1111/j.1748-7692.2003.tb01121.x
11. Baird RW, Hedrick NM, Gorgone AM, Thieleking JL, McSweeney DJ, Robertson KM, Webster DL. Population structure of island-associated dolphins: Evidence from mitochondrial and microsatellite markers for common bottlenose dolphins (Tursiops truncatus) around the main Hawaiian Islands. Mar Mammal Sci. 2012; 28:E208–E232. https://doi.org/10.1111/j.1748-7692.2011.00506.x
12. Fernández-Bellon H, Vergara-Alert J, Almagro V, Rivas R, Sánchez A, MartínezMC, Majó N, Busquets N, Ramis A. Vaccination against H5 avian influenza virus induces long-term humoral immune responses in flamingoes (Phoenicopterus spp.). Vaccine. 2016; 34:3082–3086. https://doi.org/10.1016/j.vaccine.2016.04.078 PMID:27151883
13. Johnson AR. An overview of the greater flamingo ringing program in the Camargue (Southern France) and some aspects of the species' breeding biology studied using marked individuals. Waterbirds Int J Waterbird Biol. 2000; 23:2. https://doi.org/10.2307/1522140
14. GW Howard. Conservation of the lesser flamingo in Eastern Africa and beyond: Proceedings of a workshop at Lake Bogoria, Kenya, 26th to 29th August, 1997 (IUCN Eastern Africa Programme).
15. Tavecchia G, Pradel R, Boy V, Johnson AR, Cézilly F. Sex- and age-related variation in survival and cost of first reproduction in greater flamingos. Ecology. 2001; 82:165–174. https://doi.org/10.1890/0012-9658(2001)082[0165:SAARVI]2.0.CO;2
16. Perrot C, Béchet A, Hanzen C, Arnaud A, Pradel R, Cézilly F. Sexual display complexity varies non-linearly with age and predicts breeding status in greater flamingos. Sci Rep. 2016; 6:36242. https://doi.org/10.1038/srep36242 PMID:27883016
17. Hoyo J del., Elliott A, Sargatal J, Cabot J. Handbook of the birds of the world Vol. 1: Ostrich to Ducks ed Lynx Edicions (Lynx Edicions). 1st Ed. 1992.
