# Supplementary material for: Slower rates of accumulation of DNA damage in leukocytes correlate with longer lifespans across several species of birds and mammals
Source: Aging (Albany NY). 2019 Nov 15;11(21):9829–45. doi: 10.18632/aging.102430 (PMC6874430; doi:10.18632/aging.102430)
Supplement: Supplementary Figure 1 [file aging-11-102430-s003.pdf]

SUPPLEMENTARY FIGURE

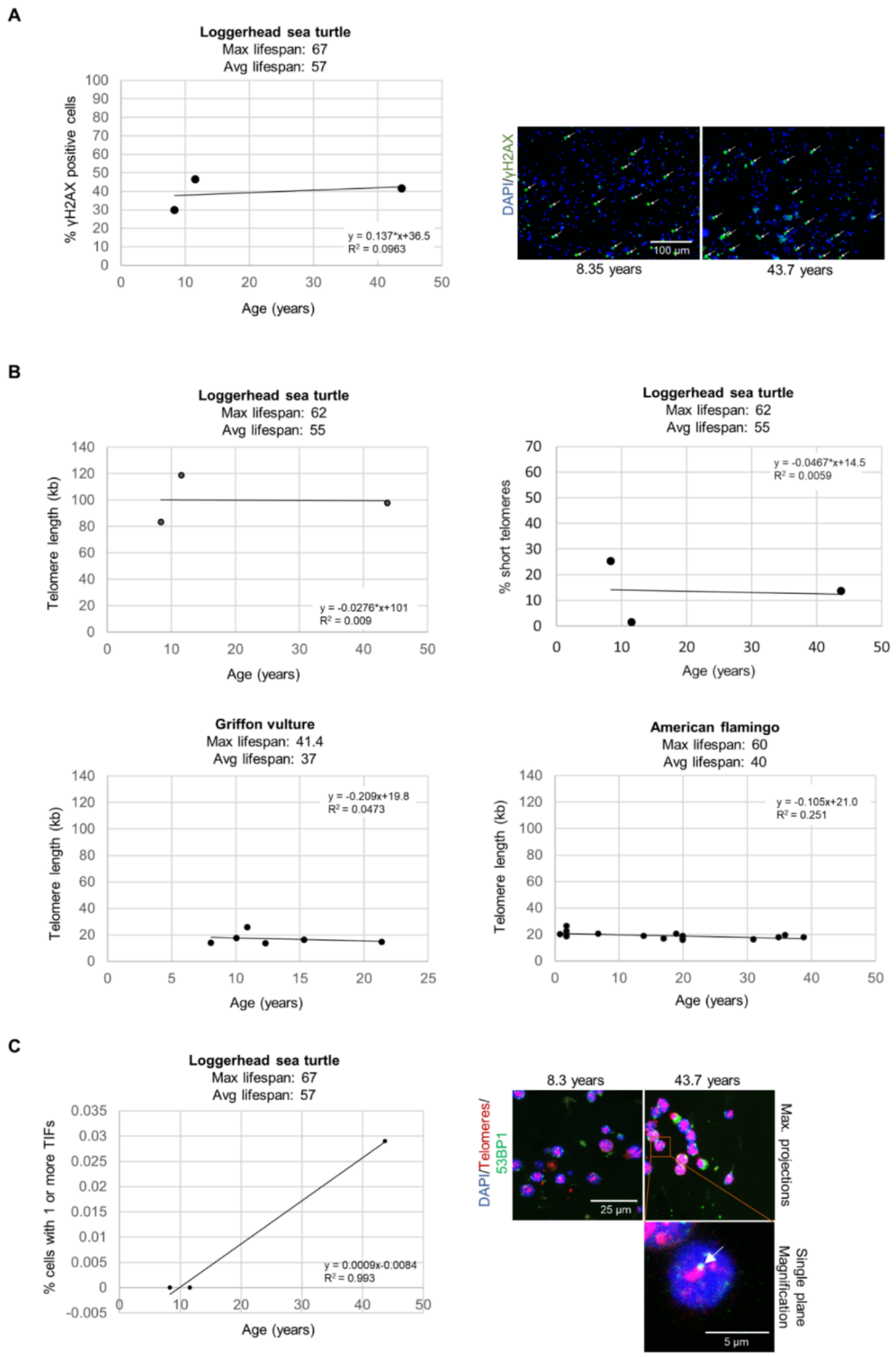

Supplementary Figure 1. DNA damage and telomere lengths measured in lymphocytes of loggerhead sea turtles. (**A**) The level of γH2AX was measured by immunofluorescence in a high-throughput manner in individuals of different ages for loggerhead sea turtles (*Caretta caretta*). Each point represents the values for a different individual. The correlation coefficient (R^2), slope (rate of γH2AX increase in % positive cells per year), and y-intercept are presented on the graphs. Representative images show cell nuclei stained with DAPI in blue and

γH2AX stain in green for a young individual and an older individual. White arrows indicate γH2AX positive cells. (**B**) Telomere measurements and percent short telomeres for the loggerhead sea turtle. The telomere measurements of the griffon vulture and American flamingo are also shown for comparison (the percent short telomeres of the griffon vultures and American flamingos are shown in Figure 3). The telomeres were measured by HT Q-FISH in individuals of different ages for loggerhead sea turtles (*Caretta caretta*), griffon vultures (*Gyps fulvus*), and American flamingos (*Phoenicopterus ruber*). Each point represents the values for a different individual. The correlation coefficient (R^2), slope, and y-intercept are presented on the graphs. The telomere length graphs for the griffon vultures and the American flamingos derive from a previous publication in our lab (1), but they are shown here for comparison with the turtles. (**C**) The percentage of cells with one or more TIFs in loggerhead sea turtles (*Caretta caretta*) is shown. The age and number of cells measured for each individual was as follows: 8.3 years: 58 cells, 11.6 years: 159 cells, and 43.7 years: 69 cells. In the representative images, the nuclei are stained blue with DAPI, the telomeres are red, and the 53BP1 stain is in green. 53BP1 staining at the very edge of nuclei was not counted as foci. The top row of the representative images shows maximum projections which are the result of taking the maximum value of several different z-planes. The magnification image is displayed for a single plane rather than a maximum projection to show colocalization of the stains. A white arrow indicates a co-localization of 53BP1 and a telomere spot (a TIF).
