# Supplementary material for: Slower rates of accumulation of DNA damage in leukocytes correlate with longer lifespans across several species of birds and mammals
Source: Aging (Albany NY). 2019 Nov 15;11(21):9829–45. doi: 10.18632/aging.102430 (PMC6874430; doi:10.18632/aging.102430)
Supplement: Supplementary Tables [file aging-11-102430-s002.pdf]

SUPPLEMENTARY TABLES

Supplementary Table 1. Telomere length, rate of telomere shortening, lifespan, and DNA damage.

Species	Telomere Length at Young Age (kb)	Rate of Telomere Shortening (bp/year)	Maximum Lifespan (years)	Average Lifespan	Rate of γH2AX increase (% positive cells/year)	Rate of increase of % short telomeres (%/year)
Goat (*Capra hircus*)	**10.4** [1]	**363** [1]	**20.8**	**16.5** [3]	**0.668**	**0.218**
Reindeer (*Rangifer tarandus*)	**19.8** [1]	**531** [1]	**21.8**	**15** [4–6]	**1.24**	**0.947**
Griffon vulture (*Gyps fulvus*)	**19.8** [1]	**209** [1]	**41.4**	**37** [5, 7]	**0.472**	**0.451**
Bottlenose dolphin (*Tursiops truncatus*)	**90.7** [1]	**766** [1]	**51.6**	**17** [8–11]	**0.115**	**0.119**
American flamingo (*Phoenicopterus ruber*)	**21.0** [1]	**105** [1]	**60**	**40** [12–17]	**0.180**	**0.394**

For each species in the study, the estimated initial telomere length (1), the rate of telomere shortening (1), the maximum lifespan, the average lifespan, the rate of DNA damage, and the rate of increase of % short telomeres is shown. The maximum lifespans were obtained from the AnAge database (2), and the average lifespans were obtained from various sources as referenced in the table.

Supplementary Table 2. Data table used for multivariate linear regression.

Species	Rate of γH2AX increase (% positive cells/year) (log)	Rate of increase of % short telomeres (%/year) (log)	Average lifespan (years) (log)	Maximum lifespan (years) (log)
Goat (*Capra hircus*)	-0.175	0.314	1.22	1.32
Reindeer (*Rangifer tarandus*)	0.0934	-0.02365	1.18	1.34
Griffon vulture (*Gyps fulvus*)	-0.370	-0.346	1.57	1.62
Bottlenose dolphin (*Tursiops truncatus*)	-0.939	-0.924	1.23	1.71
American flamingo (*Phoenicopterus ruber*)	-0.745	-0.405	1.60	1.78

The log values of all of the datapoints were used for the regression. The input variables of rate of γH2AX increase (% positive cells/year) and rate of increase of % short telomeres (%/year) were fit to either the average lifespan or the maximum species lifespan.

Supplementary Table 3. Multivariate regression results for average lifespan.

	Estimate	Standard error	t-value	Pr(>|t|)
(Intercept)	1.27	0.193	6.57	0.0224 (*)
Rate of γH2AX increase (% positive cells/year) (log)	-0.283	0.590	-0.480	0.679
Rate of increase of % short telomeres (%/year) (log)	0.110	0.535	0.206	0.856

The estimate, standard error, t-value, and p-value of each variable is presented.
Residual standard error: 0.269 on 3 degrees of freedom
Multiple R-squared: 0.152, Adjusted R-squared: -0.695
F-statistic: 0.180 on 2 and 2 DF, p-value: 0.848

Supplementary Table 4. Multivariate regression results for maximum lifespan.

	Estimate	Standard error	t-value	Pr(>|t|)
(Intercept)	1.38	0.0694	19.9	0.00252 (**)
Rate of γH2AX increase (% positive cells/year) (log)	-0.251	0.212	-1.18	0.358
Rate of increase of % short telomeres (%/year) (log)	-0.180	0.192	-0.938	0.447

The estimate, standard error, t-value, and p-value of each variable is presented.
Residual standard error: 0.0966 on 2 degrees of freedom
Multiple R-squared: 0.875, Adjusted R-squared: 0.750
F-statistic: 6.99 on 2 and 2 DF, p-value: 0.125
